# The Institution Food Controller

**Published:** 1917-09-29

**Authors:** 


### FOOD IN WARTIME.

### The Institution Food Controller.

The stirring of a new spirit of economy among English people has speedily revealed the extent to which ordinary foodstuffs were wasted as a matter of course in days gone by. The reckless use of sugar, bread, and meat in almost every household in the kingdom has now received a severe check, but it would be idle to pretend that a radical alteration has taken place in our national habits of profusion. Even where conscience curbs the appetite, waste often goes on simultaneously, and many who rise but partially satisfied from their meal would be horrified if they could watch the misuse of the remains of their loaf or joint when it returned to the kitchen.

The need is for a "food controller" in every household. In the private house where the wife chooses, buys, stores, cooks, and dispenses every article of diet economy often rises to a delicate art. It has the grace ascribed to the original "loaf-giver" or lady. It tolerates nothing coarse or unsound. It produces that effect of choice selection combined with skilled manipulation characteristic of the good chef. Everything is far too good to be wasted, and the cost of good food when waste has been eliminated is astonishingly more moderate than that of bad.

### Co-ordination in the Commissariat.

Managers of institutions who desire to get the cost of food reduced must study to introduce the principle of one "food controller." If the system be faulty in this respect there can be no certainty that all the savings conscientiously achieved by the consumers are not dissipated in the larder or at some other stage in the cycle through which foodstuffs must pass. Complete co-ordination demands that the process of selecting and ordering the food, of keeping it in good condition in the interval before it is used, of building the menu, and of preparing the food for table in accordance with it, should be under one authority. In by far the greater number of institution households three or even four persons divide up these duties without any attempt at mutual consultation. The prescribed dish for the day may be roast beef, for instance. Accordingly so many pounds of beef arrive, and the cook's only duty is to roast or bake in accordance with orders from above. But it is quite possible that the beef may prove unsuitable for the purpose; too fresh to be cooked at all, too tough or stringy to be roasted, with big bones or lumps of suet attached, or with flaps which when roasted are almost pure waste. In an establishment where want of co-ordination prevails, the cook simply obeys orders and roasts the meat as it is sent in. The matron or whoever carves may complain that the meat is tough, or dried up, or very fat, or that it seems all bone. But she has not selected the meat, nor has the cook, and hence the complaints are received with disfavour and practically ignored by the caterer.

### The Sunday Roast Beef.

Supposing, however, that the "food controller," whom we may assume to be a trained housekeeper with a good knowledge both of buying and cooking, be charged with the duty of controlling the supplies. She will know before she makes her purchases that the surplus from a roast-beef day will go some way towards providing a second day's dinner, and she will take into account the quantities required for both days. Her selection will be made early in the morning preceding the day when the meat is to be roasted—on Saturday, should Sunday roast-beef be the rule. The open market will be her preference, but if there be none available she will select from a good firm whose interest it will be to give satisfaction. She will order twice as much as is required for one day's dinner, or at the rate of about ½ lb. (according to present rations) per head. On delivery the meat will be hung in an airy meat-larder, provided with a meat-block, chopper, saw, and sharp knives. There the joints will be boned, and very carefully trimmed of every scrap of flesh or gristle, which, when roasted, would turn to waste. The suet will be taken off, lightly dusted with flour, and set aside for use. The trimmings and outer edges of the joints will be stewed down and set aside for the fat to be skimmed off when cold. The bones and rough trimmings will go into the stock-pot. The main joints, deftly rolled and firmly secured, will be served roasted on Sunday, and it is not too much to say that the cook should be conscious of failure if a single mouthful remain unconsumed on any plate. To this end the "food controller" will take good heed that the carving-knives are brought into first-rate condition on the Saturday, and that the best carver the establishment can boast is selected for the delicate task of covering an entire plate with 3 oz. of finely-cut meat. In order to carve well the beef must be sufficiently roasted, for nothing cuts to greater waste than half-raw meat. It must not, however, be subjected to a heat which chars the outer edges and dries the juices.

### The Next Day's Dinner.

The remains after the Sunday dinners have been consumed will be as follows: First, there will be one or more joints of rolled beef (according to the size of the household) uncut; next there will be the pieces (stewed down and ready for use) which were trimmed from the joints because unfit to be roasted; thirdly, there will be suet; and, lastly, the bones and gristle will have yielded material for soup. The menu for Monday will, then, be cold roast beef, with rissoles or camp pie, or hotpot made from the pieces as an alternative, followed by a pudding in which suet is an ingredient. Where it is important to save labour and economise fuel, sufficient beef could be ordered to allow of the hot roast beef on Saturday; cold roast beef (allowed to go cold before being cut) on Sunday, and the rissoles or camp pie preceded by soup on Monday. The advantage of this arrangement is that the "food controller" knows exactly what she is going to do with each ounce of meat, fat, and bone which she purchases, and she can get better value than would be possible supposing the joints were bought without reference to the needs of the ensuing day. Moreover, should she find that joints suitable for roasting are not to be had at the price she desires to give, she can rearrange the menu to suit the vagaries of the market in war-time, and serve the beef braised or boiled. The important point is that one person should retain control of the food supplies from the moment of purchase till they pass into the mouths of the consumers, and guard against waste at every step of the road. But the person selected for this office must be thoroughly trained for her work and adequately paid.

☛ Previous articles appeared February 24, March 10, 24, and 31, April 14 and 21, May 5, 12, and 19, June 9 and 16, and September 1.

